# Rosiglitazone improves the natriuretic response to atrial natriuretic peptide in rats with experimental congestive heart failure: possible involvement of a post-guanylate cyclase mechanism

**DOI:** 10.1186/1471-2210-11-S1-P29

**Published:** 2011-08-01

**Authors:** Ilia Goltsman, Elena Ovcharenko, Aaron Hoffman, Giora Feuerstein, Zaid Abassi, Joseph Winaver

**Affiliations:** 1 Department of Physiology and Biophysics, Faculty of Medicine, Technion-IIT, Haifa, Israel

## Background

Congestive heart failure (CHF) in patients and experimental animals is characterized by renal retention of salt and water and a blunted natriuretic/diuretic response to atrial natriuretic peptide (ANP). Recently, we reported that chronic treatment with the PPARγ-agonist rosiglitazone (RGZ) improved the natriuretic and diuretic responses to extracellular fluid volume expansion in rats with aorto-caval fistula, an experimental model of volume-overload CHF [1]. In the present study we explored whether RGZ improves also the natriuretic/diuretic response to ANP in rats with experimental CHF. In addition, we evaluated the effects of the drug on ANP-mediated cGMP signalling in the kidney.

## Methods

CHF was induced in male Sprague-Dawley rats by surgical formation of a fistula between the aorta and inferior vena cava (1.2 mm O.D.). The natriuretic/diuretic responses and urinary cGMP (UcGMP) excretion in response to rat ANP (15 and 50 µg/kg/hr, low and high doses, respectively) were assessed by clearance methodology in CHF rats following 2 weeks of RGZ (30 mg/kg/day, p.o.) or vehicle (Veh) treatment, and in sham-operated controls (N=6-10). The capacity of isolated glomeruli and collecting ducts to generate cGMP in-vitro in response to ANP (10^-11^-10^-6^M, in the presence of 1.0 mM IBMX) was tested. Urinary and tissue cGMP levels were measured using ELISA. Moreover, to screen for relevant gene expression targets of RGZ (renal cortex and medulla samples, N=3 in each group), quantitative real-time PCR was performed using Taqman^®^ array plates testing 32 selected genes related to ANP/cGMP signalling and controls.

## Results

**Figure 1:**
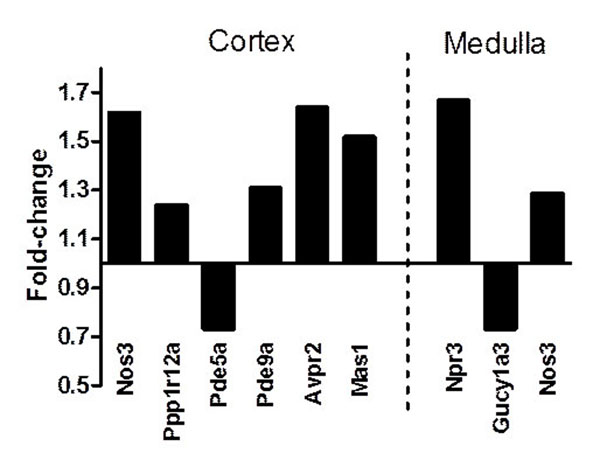
Renal RGZ-regulated genes related to ANP signalling and Na^+^ transport in rats with CHF. Fold-change represent comparison of CHF+RGZ vs. CHF+Veh (N=3 each). P-values for all comparisons are lower than 0.07. Averaged values for Gapdh, Ppia and Gusb were used as normalizers and the sham-control group served as the calibrator. Nos3, endothelial NO synthase; Ppp1r12a, myosin phosphatase-targeting subunit 1; Pde5/9a, cGMP-specific phosphodiesterases; Avpr2, Arginine vasopressin receptor 2; Mas1, Ang(1-7) receptor Mas; Npr3, Natriuretic peptide receptor C; Gucy1a3, soluble guanylate cyclase α1-subunit.

CHF rats exhibited a markedly blunted natriuretic response to ANP administration. RGZ treatment significantly improved the natriuretic/diuretic action of ANP in CHF rats compared with Veh treatment, as represented by urinary sodium excretion rate (Control+Veh: 1.3±0.3 to 9.2±2 µEq/min, CHF+Veh: 0.3±0.16 to 2.3±0.7* µEq/min, CHF+RGZ: 2.3±0.7 to 9.5±1.9^†^ µEq/min, baseline to low dose ANP values, *p<0.05 vs. Control+Veh, ^†^p<0.05 vs. CHF+Veh). However, UcGMP excretion was similarly increased in response to ANP and did not differ between CHF rats treated with RGZ or Veh (Control+Veh: 14±3 to 129±26 pmol/ml, CHF+Veh: 65±10 to 127±15 pmol/ml, CHF+RGZ: 47±10 to 143±22 pmol/ml, GFR-normalized values, p=NS). Also, RGZ treatment did not alter the capacity to generate cGMP in response to ANP in isolated renal tissues. Finally, preliminary gene expression study revealed several genes related to renal ANP/cGMP signalling and Na^+^ transport regulation that were significantly altered by RGZ treatment in rats with CHF (see Figure 1).

## Conclusion

In rats with experimental CHF, chronic RGZ treatment enhances renal ANP-induced natriuresis, probably at a step beyond cGMP generation. Furthermore, the data reveal several gene expression targets, including members of the phosphodiesterase family, eNOS and Mas receptor, that may be involved in the improvement in ANP-induced natriuresis.
